# Comparison of volatile aroma compounds and consumer perception between domestic and imported milk in Korea

**DOI:** 10.1007/s44463-025-00018-9

**Published:** 2026-04-02

**Authors:** Min Kyung Park, Seyeong Park, Gyeonghye Yoon, Cho-Long Lee, Han Sub Kwak

**Affiliations:** 1 Food Processing Research Group, Korea Food Research Institute, 245, Nongsaengmyeong-ro, Iseo-myeon, Wanju-gun, 55365 Korea; 2 Department of Nutritional Science, Ewha Womans University, Seoul, 03760 Korea; 3 Major in Food Biotechnology, University of Science and Technology, Daejeon, 34113 Korea

**Keywords:** Milk, Imported milk, Volatile aroma compound, Consumer acceptance, Country of origin

## Abstract

This study analyzed the volatile aroma compounds and compared consumer acceptance of domestic and imported milk in Korea through blind and informed sensory evaluations. Seven milk samples, including four domestic (two refrigerated and two sterilized) and three sterilized milk imported from Germany, Poland, and Australia, were analyzed to compare volatile aroma components, sensory profiles, and influence of extrinsic factors such as brand, packaging, price, and country of origin (COO). The analysis of volatile aroma compounds revealed that imported milk had a relatively higher total level of volatile aroma compounds compared to domestic milk. Additionally, the aroma characteristics of imported milk were primarily influenced by stronger dairy-related flavors, such as cheese and butter, as well as pungent sulfur-derived compounds, compared to domestic milk. Consumer tests revealed that both acceptance and familiarity for Korean milk were high and these ratings further increased in the informed test due to strong consumer trust in domestic products. Although imported milk samples were competitively priced, their acceptance declined slightly more when the package and brand information were presented. Price had a minimal influence on consumer preference, and intrinsic factors such as flavor and aroma played dominant roles. These results indicate that, over time, Koreans have become accustomed to the milder dairy-related flavors of domestic milk; therefore, imported milk, which has strong dairy-related aroma characteristics, was less accepted and less familiar to them.

**Supplementary Information:**

The online version contains supplementary material available at 10.1007/s44463-025-00018-9.

## Introduction

In the Republic of Korea, the consumption of dairy products has shown a rising trend from 63.9 kg in 2001 to 86.1 kg in 2021, largely driven by the growing demand for cheese, butter, and cream (KDI, 2022). However, milk consumption has declined over the past two decades from 36.5 kg to 21.0 kg in the same period (KDI, 2022). This decline can be attributed to several factors, including increasing milk price, declining birth rate, and rising popularity of plant-based milk. Despite the decline in milk consumption, most milk consumed is produced domestically using raw milk sourced within the country. However, raw milk production has decreased from 2.34 million tons in 2011 to 2.03 in 2021 (KDI, 2022), prompting an increase in the import of dairy products, particularly sterilized milk, because of rising domestic milk prices (KCD, 2024, KDI, 2022).

The majority of milk distributed in Korea is refrigerated and locally produced, which must be consumed before the end of its short shelf life, which is 14 days. However, increasing production costs have made Korean milk some of the most expensive globally, leading to a surge in the importation of sterilized milk, which has a longer shelf life and is easy to distribute (GlobalProductPrices, 2024). Imported sterilized milk is now more accessible to Korean consumers through business–business channels and online platforms, offering a more cost-effective alternative to domestic refrigerated milk. In addition to economic factor, imported milk offers diversity to the Korean milk market in terms of flavor and taste, which may differ because of the varying environmental and agricultural conditions of cattle farming in different countries (Chi et al., 2023; Clarke et al., 2019). However, Korean consumers’ preferences for imported milk versus domestic milk remain unclear.

Country of origin (COO) is a well-established factor that influences food choice and acceptance. Several studies have demonstrated that consumers often display ethnocentric tendencies, favoring domestically produced goods over imported ones, a phenomenon observed in Hungary (Garai-Fodor & Popovics, 2021), Chile (Schnettler et al., 2017), Nigeria (Kilders et al. 2021), and Norway (Vabø et al., 2017). Moreover, the COO of food products can significantly increase consumer preference, particularly with strong ethnocentric attitudes. For example, in Vietnam, Le et al. (2017) found that the COO was an indirect factor in generating product attitudes toward powdered milk; however, consumers with higher ethnocentrism levels were less likely to purchase imported powdered milk. Similar findings have been reported in Korea. For example, Kwak et al. (2019) indicated that Korean consumers demonstrated a higher overall acceptance of pan bread made from domestic wheat flour when the COO was disclosed compared with that when unknown. These results showed that the COO can play a role in making consumer attitudes and choices, even for products as common as bread.

Previous studies on dairy products have revealed that the COO influences consumer preference. Gedikoglu and Parcell (2014) revealed consumer preferences between domestic and imported artisan cheese, presenting how the perception of the COO is one of key factors in decision-making. Similarly, in China, Zhang et al. (2021) reported that Chinese consumers were willing to pay more for imported ultrahigh temperature (UHT) milk from Australia and New Zealand, considering the dairy products from these countries to be premium due to the perceived unreliability of domestic dairy products. While there have been studies on the effect of the COO on consumer preferences in various food products, studies on its influence in the Korean milk market is lacking. Furthermore, while imported sterilized milk offers advantages such as affordability and extended shelf life, it also presents potential disadvantages, including differences in flavor and aroma due to variations in heat treatment and storage conditions. Some studies suggest that sterilized milk may have a slightly altered taste profile compared to fresh milk (Clarke et al., 2019). Given these differences, it is essential to understand whether Korean consumers perceive these variations in sensory attributes and whether COO influences their evaluations.

In the Republic of Korea, where domestically produced milk prices continue to rise and the volume of imported milk increases, understanding consumer preferences for domestic versus imported milk is necessary. Given the increasing market presence of imported milk and potential differences in flavor and taste, this study aims to compare consumer evaluations and conduct a volatile aroma compound analysis of domestic and imported milk. For the sensory evaluation, this study intends to determine how the package, including COO and price of milk, affects the evaluation of taste and aroma through the blind and informed taste tests.

## Materials and methods

### Materials

The seven milk samples used in the experiment are shown in Table 1. Milk from the company with the highest market share and from another company producing milk in a different region were selected. Two domestically produced milk samples (KEC1 and KEC2) were pasteurized using UHT and distributed under refrigeration. Their shelf life was 14 days. The other two domestically produced milk samples (KER1 and KER2) were sterilized in UHT, distributed at room temperature, and had a 6-week shelf life. The shelf life of domestically produced milk is regulated by the Korean government. For imported milk samples, three milk samples produced in different countries were selected and purchased through online market. These samples from Germany (DER), Poland (PLR), and Australia (AUR) were sterilized in UHT with a 1-year shelf life. Samples were purchased from local grocery stores or online shopping sites. All samples were kept in a refrigerator (4 °C) after purchase and served within the expiration date.

**Table 1:** Milk sample information

Sample	Origin	Distribution method	Pasteurization temperature (°C)/ time (s)	Shelf life	Price (won/L)
KEC1	Korea	Refrigerated	above 130/2	10 days	2980
KEC2	Korea	Refrigerated	above 130/2	10 days	2975
KER1	Korea	Ambient	132–150/3	6 weeks	2480
KER2	Korea	Ambient	above 135/3	6 weeks	2730
AUR	Australia	Ambient	141–145/2–6	1 year	2700
PLR	Poland	Ambient	135–145/3	1 year	1620
DER	Germany	Ambient	130–140/2–4	1 year	2280

### Volatile aroma compound analysis

2-Methyl-heptanone was obtained from Sigma-Aldrich (USA) as an internal standard. The solid-phase microextraction (SPME) system was equipped with a divinylbenzene/carboxen/polydimethylsiloxane fiber (50/30 µm, Supelco, Bellefonte, PA, USA) coupled with a gas chromatography–mass spectrometry (GC–MS) system. The milk sample (3.0 mL) was placed into an amber vial (20 mL) with the internal standard (2 µL, 100 µg/mL of 2-methyl-heptanone in methanol). It was incubated in the amber headspace vial for 30 min at 40 °C. Then, the volatile aroma compounds were absorbed for 30 min at 40 °C using an SPME fiber.

GC–MS analysis was performed using a 7890 A Agilent GC system (Agilent Technologies, Santa Clara, CA, USA) coupled with a 5975 C MSD mass detector (Agilent Technologies). A DB-WAXUI capillary column (30 m × 0.25 mm i.d. × 0.25-µm film thickness, J&W Scientific, Folsom, CA, USA) was used for the separation of the volatile aroma compounds. Helium (99.999%) was used as the carrier gas at a flow rate of 0.8 mL/min. The temperatures of the injection port and transfer line were 230 °C and 250 °C, respectively. The oven temperature was maintained at 40 °C for 5 min and increased to 200 °C with a rate of 4 °C/min, which was then maintained for 2 min. The volatile aroma compounds were obtained by the electron impact ion source at 70 eV and scanned in the range of 35–350 atomic mass units.

The volatile aroma compounds were quantified by comparing their peak areas with that of an internal standard. The identification was also confirmed by comparing the retention times and mass spectral data with those of authentic standard compounds or the data in a commercial GC–MS library (Wiley 7.0). The retention indices of the volatile aroma compounds were calculated with n-alkanes (C_7_–C_30_ Saturated Alkanes, Supelco, Bellefonte, PA, USA).

One-way analysis of variance (ANOVA) was performed using Duncan’s multiple range test to compare the significant differences (*p* < 0.05) between samples using IBM SPSS Statistics for Windows version 20.0 (IBM Corp., Armonk, NY, USA). Principal component analysis (PCA) and partial least squares discriminant analysis (PLS-DA) were performed using SIMCA 17.0 (Umetrics, Umea, Sweden).

## Consumer test

### Participants

The participants (*n* = 120) were recruited from the local residents of Jeonju-si and Wanju-gun, Republic of Korea, where approximately 700,000 people reside. They participated voluntarily in the experiment for two consecutive days and had no health problems drinking milk. The consumer test was conducted in the sensory facility at the Korea Food Research Institute (KFRI; Wanju-gun, Republic of Korea). Finally, 114 participants completed two consumer tests. This study was approved by the KFRI Institutional Review Board (Approval no. KFRI 2023-11-001-001).

### Sample Preparation and test procedure

Milk samples were prepared one day before each testing date. Approximately 30 g of each sample was poured into transparent plastic cups (70 mL, Dongyang Kaida Plastics Co., Ltd, Zhejiang, China) coded with random three-digit numbers. Samples were sealed using Parafilm and placed in a refrigerator (4 °C) a day before testing. The samples were served under a slightly chilled condition: 8 °C ± 2 °C based on the pre-experiment. Participants evaluated the samples in individual sensory booths and rated their liking and sensory profiles using a computerized sensory data collection system (Compusense Cloud, Compusense Inc., Guelph, Canada). After tasting the milk samples, participants evaluated overall liking and other liking parameters, including color, flavor, aroma, and mouthfeel, on a 9-point scale (1, extremely dislike; 5, neither like nor dislike; and 9, extremely like). The sensory profile of each sample was measured using the rate-all-that-apply (RATA) method. The RATA question consisted of 24 attributes (3 for appearances, 5 for aromatics, 11 for flavor, and 5 for mouthfeel) with a slight modification from the method published by Heo et al. (2023). Twenty-five emotional responses (0, none; 1, weak; 2, medium; 3, strong) for each milk sample were assessed using EsSense25^®^, a shorter version of the EsSense Profile^®^ (Nestrud et al., 2016). Participants answered the RATA and emotion questions using a 3-point scale (1, week; 2, moderate; and 3, strong) only when they perceived each attribute. The presentation order of the seven samples followed a Williams Latin square design. Cracker (Carr’s Table Water Cracker, Pladis-uk-carlisle, London, UK) and water were served with milk for palate cleansing between samples.

The procedure of the second experiment was identical to the first, except that actual 1-L packages of each sample, along with their prices, were presented to the participants. The participants were asked to look over the package first when they received the samples.

### Statistical analyses

For the liking and familiarity data, one-way analysis of variance (ANOVA) was performed for each test; blinded and informed test. Tukey’s honest significant difference test was employed to discern significant differences across the samples at *p* < 0.05. Paired t-test was conducted to compare significant differences in each liking attribute and familiarity at *p* < 0.05. In the data matrices of the RATA method, unchecked attributes were replaced with zero as part of data preprocessing (Meyners et al., 2016). For each test condition, the mean ratings of the sensory attributes for every sample were computed. PCA was conducted to present the relationships between attributes and samples in each test condition (blind vs. informed). Partial least square (PLS) regression was conducted using volatile chemical compounds as the independent variable and sensory evaluation results as dependent variables. All data analyses were conducted using XLSTAT version 2024 (Lumivero, Denver, CO, USA).

## Results

### Comparison of volatile aroma compounds in domestic and imported milk

A total of 42 volatile aroma compounds were identified in the milk samples. Table S1 lists the identified volatile aroma compounds and their contents. Ketones and benzenes made up the largest portion of the total volatile aroma compounds in milk, as in the previous study (Natrella, Gambacorta, De Palo, Maggiolino, & Faccia, 2020). Specifically, acetone accounts for 50%–70% of the total volatile aroma compounds in milk, and this dominance has been reported in the literature (Clarke et al., 2019; Toso, Procida, & Stefanon, 2002). Acetone, which has a pungent and solvent odor (Hillert, Musabasic, Berglund, Ciumas, & Savic, 2007), is related to the nutritional status of cows and is more prevalent in feeds, such as hay and wheat (Villeneuve et al., 2013).

**Fig. 1:**
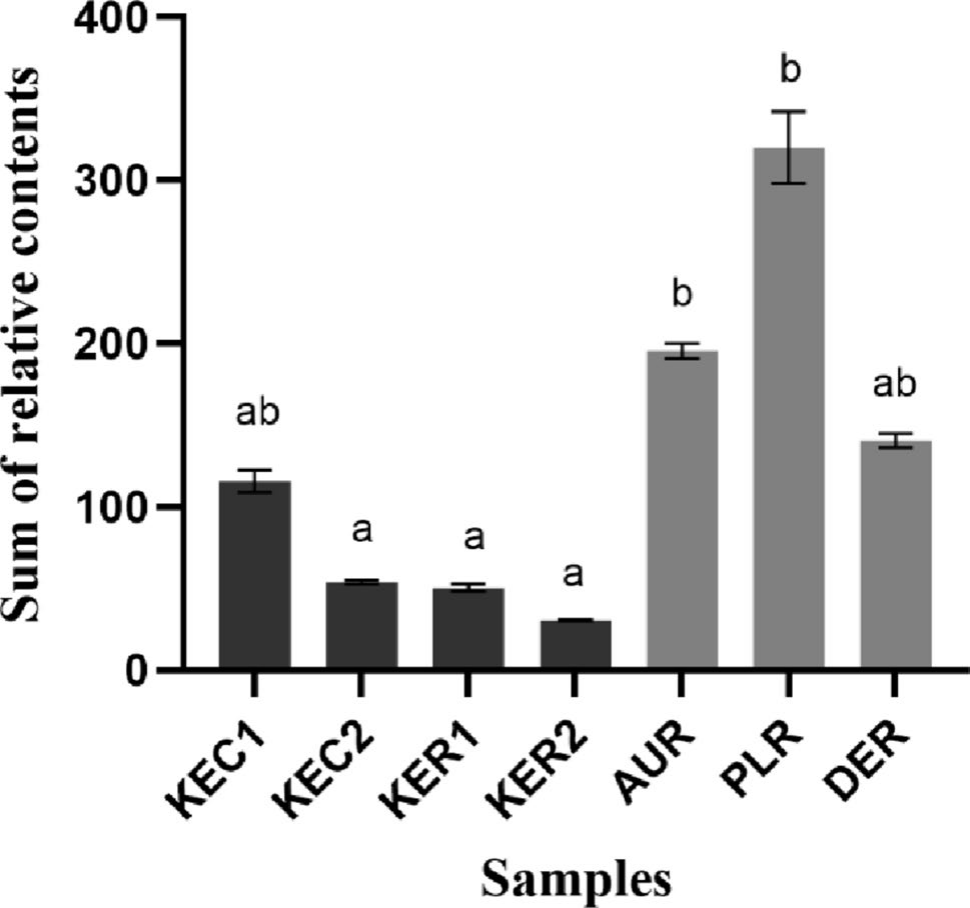
The sum of the relative contents of volatile aroma compounds derived from each milk sample. Different letters in the bar graph indicate significant differences by Duncan’s multiple-range test at *p* < 0.05

A difference was noted in the quantitative sum of all the volatile aroma compounds between the domestic (KEC1, KEC2, KER1, and KER2) and imported milk (AUR, PLR, and DER) samples. In Fig. 1, domestic milk samples contained quantitatively fewer volatile aroma compounds than the imported ones. In particular, AUR and PLR contained significantly higher levels of volatile aroma compounds than the other milk samples. The major detected volatile aroma compounds consisted of fatty acid-derived ketones and benzene derivatives. In the imported milks, fatty acid-derived ketones were predominant, whereas benzene derivatives were more prevalent in the domestic milks. It might be explained that differences in the chemical composition of milk may influence the formation of volatile aromatic compounds. In a previous study (Walker et al., 2004), dairy farming methods, feed composition, and grazing practices influence the fat content and composition of milk.

**Fig. 2:**
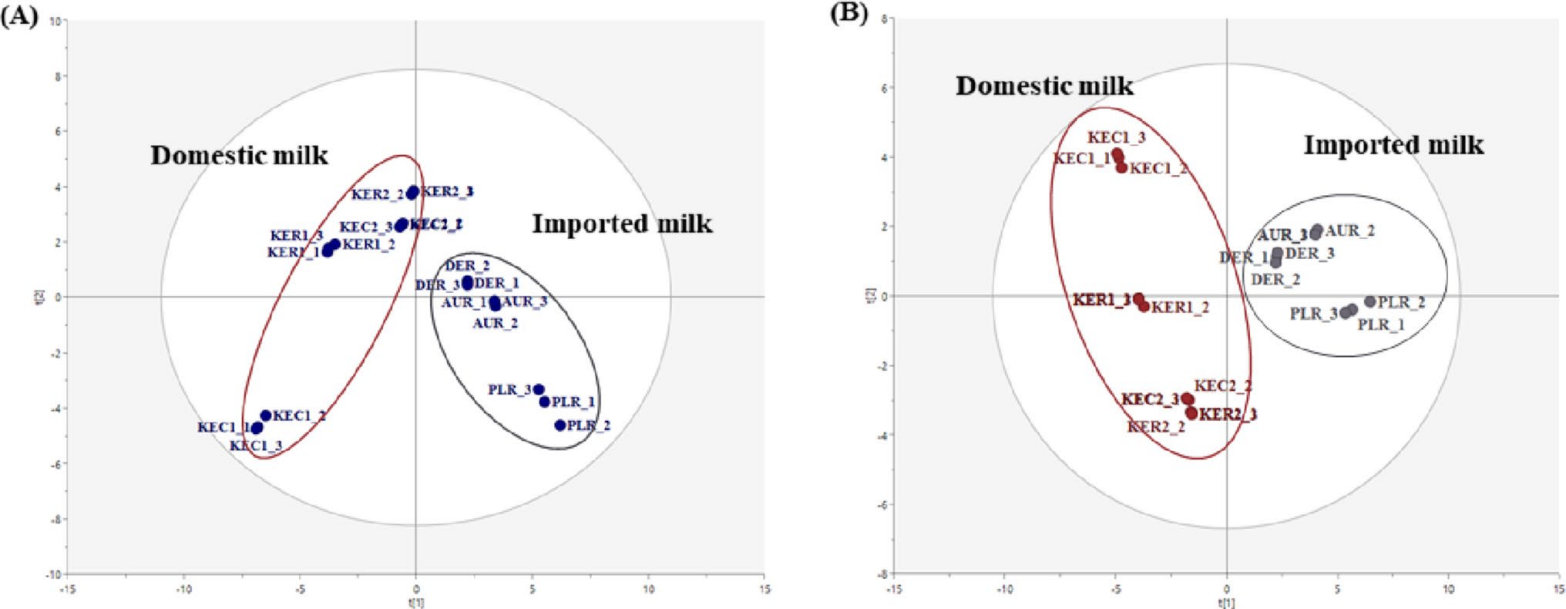
Score plots of the PCA (**A**) and PLS-DA (**B**) based on the volatile aroma profiles

The score plots of the PCA and PLS-DA based on the volatile aroma compounds derived from the milk samples are shown in Fig. 2. Multivariate statistical analyses, such as PCA and PLS-DA, were performed to confirm the similarities or differences in the volatile aroma compound profiles of samples and identify the major volatile aroma compounds contributing to the differences in the volatile aroma compound profiles between the domestic and imported milk. The distance between samples in the score plot reflects the similarity in their volatile compound profiles, with closer samples indicating more chemically similar compositions. The PCA and PLS-DA models were good for the prediction, and the domestic and imported milk samples were clearly separated into two groups by PC1 in both PCA and PLS-DA, indicating a significant difference in the volatile aroma compound profiles of the two groups. The PCA model explained 60% of the total variance, with R^2^X at 0.997 and Q^2^ at 0.988. The suitability of the PLS-DA models was evaluated with a permutation testing to check the degree of overfitting (R^2^ = 0.151 and Q^2^ = − 0.542). Based on the PLS-DA models, thresholds of VIP > 1.0 and |p(corr)| > 0.8 were chosen to find the differential features. Table S2 presents the main volatile aroma compounds that contribute to distinguishing imported milk from the domestic one in a multivariate statistical model. In total, nine volatile aroma compounds were selected, and more compounds contributed to the relatively characteristic aroma in imported milk. The imported milk group was characterized by volatile aroma compounds with distinctive odor, such as cheese and fruit aromas, representing the group’s characteristics. Methyl ketones, including propan-2-one, butan-2-one, pentan-2-one, heptan-2-one, and nonan-2-one, accounted for the major selected volatile aroma compounds contributing to the flavor of imported milk. These compounds are primarily formed from lipid oxidation and are known as key compounds in the formation of cheese and milk (Jolly & Kosikowski, 1975; Lan et al., 2025). Most sulfur-containing volatile aroma compounds have low thresholds and strong odors (Shankaranarayana et al., 1974). Methylsulfonylmethane, a sulfur-containing compound with a vegetable odor description, was identified as a major volatile aroma compound contributing to the aroma characteristics of imported milks. The results demonstrated that the imported milk group has relatively more distinctive and stronger aromas than the domestic milk group.

Milk produced in different regions or countries may vary in flavor and aroma due to factors such as cows’ diet, environmental conditions, and processing methods. Cows that graze on fresh grass produce milk with higher concentrations of volatile aroma compounds such as terpenes and carotenoids, imparting grassy and creamy notes. In contrast, cows that feed on grain or total mixed rations may produce milk with a different sensory profile, including more fatty acids and less distinct aromas (Chi et al., 2023; Clarke et al., 2019). These differences are also influenced by the biodiversity of pastures, feed quality, and rearing climate of the cows (Delaby et al., 2020; Gauly & Ammer, 2020; White, 2020). Furthermore, processing methods such as pasteurization and UHT treatments can alter the milk’s flavor by affecting the levels of volatile aroma compounds. For example, UHT-treated milk may have caramel-like or cooked flavors due to Maillard reactions during heating (Pereda et al., 2008). Volatile aroma compounds such as fatty acids, aldehydes, and ketones, which are influenced by diet and processing, are significant in the sensory properties of milk (Vargas-Bello-Pérez et al., 2018; Caputo et al., 2015). The differences in the flavor components of milk can be ascribed to the variations in the dairy cattle raising environments or manufacturing processes between Korea and the countries producing the imported milk used in this study -Germany, Poland, and Australia.

### Demographic and behavioral data of the participants

The demographic and behavioral data regarding milk consumption are presented in Table 2. The study population predominantly consisted of female participants (78.1%) and was primarily composed of individuals in their 20 s (37.7%) and 30 s (36.0%), with fewer participants in their 40 s (21.1%) and 50 s (5.3%). Occupations varied, with salaried workers (34.2%), students (30.7%), and housewives (27.2%) representing the majority. Most of the participants hold university (43.0%) or graduate school (38.6%) degrees.

**Table 2:** Demographic profiles and responses to behavioral questions about milk from participants in the sensory evaluation

Variables	Categories	Percentage (%)
Demographic questions		
Gender	Male	21.9
	Female	78.1
Age	20s	37.7
	30s	36.0
	40s	21.1
	50s	5.3
Occupation	Student	30.7
	Housewife	27.2
	Salaried worker	34.2
	Self-employed	0.9
	Unemployed	2.6
	Others	4.4
Education	High school	10.7
	College	7.9
	University	43.0
	Graduate school	38.6
Family monthly income (won)	Below 3 million	24.6
	3–5 million	33.3
	5–7 million	27.2
	above 7 million	14.9
Questions for consumption and purchase of milk		
Consumption experience of regular refrigerated milk	Yes	98.2
	No	1.8
Consumption experience of shelf-stable UHT milk	Yes	66.7
	No	33.3
Consumption experience of domestic milk	Yes	100.0
	No	0.0
Consumption experience of imported milk	Yes	15.8
	No	84.2
Purchase experience of regular refrigerated milk	Yes	99.1
	No	0.9
Purchase experience of shelf-stable UHT milk	Yes	71.9
	No	28.1
Purchase experience of domestic milk	Yes	100.0
	No	0.0
Purchase experience of imported milk	Yes	12.3
	No	87.7
Type of milk mainly purchased	Refrigerated milk	86.8
	Shelf-stable milk	13.2
Origin of milk mainly purchased	Domestic	100.0
	Imported	0.0

Consumption and purchasing behaviors indicated a strong preference for regular refrigerated milk (Table 2), and nearly all participants had consuming (98.2%) and purchasing (99.1%) experiences. In contrast, shelf-stable UHT milk was less commonly consumed (66.7%) and purchased (71.9%). Notably, each participant had consumed and purchased domestic milk, whereas a minority reported consumption and purchase experience with imported milk at 15.8% and 12.3%, respectively. Furthermore, refrigerated milk was the predominant choice (86.8%) compared with shelf-stable milk (13.2%), domestic milk was exclusively favored, and preference for imported milk was not reported. These findings highlight a clear consumer preference toward domestic refrigerated milk, underscoring the significant influence of milk origin and storage type on participants’ purchasing decisions.

### Comparison of liking and familiarity between blind and informed tests

**Fig. 3:**
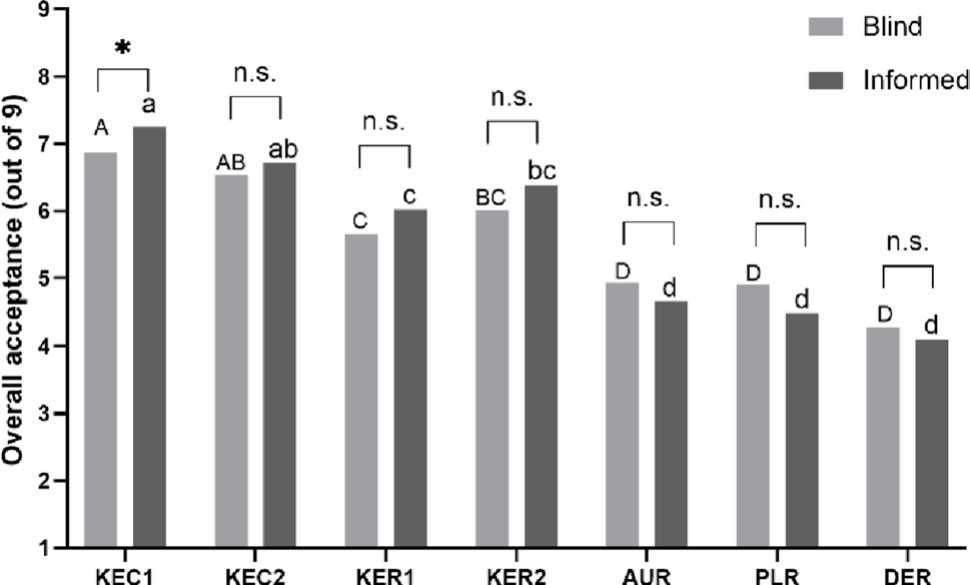
Comparison of mean overall acceptance ratings of the blind and informed tests of milk samples. Uppercase letters indicate significant differences (*p* < 0.001) among samples in the blind test, whereas lowercase letters indicate significant differences in the informed test. * means a significant difference between the blind and informed tests for each same sample at *p* < 0.05. n.s. meant non-significant

The overall liking ratings in the blind and informed tests are provided in Fig. 3. Significant differences were noted across the samples (*p* < 0.05), and the same mean separation pattern was found between the blind and informed tests. Domestically produced refrigerated milk samples (KEC1 and KEC2) received the highest liking ratings, followed by domestically produced sterilized milk samples (KER1 and KER2). Imported sterilized milk (DER, PLR, and AUR) samples received significantly lower liking ratings compared with domestic ones (*p* < 0.05). In the change of overall liking depending on whether information was provided, liking scores for domestic milk samples generally tended to increase when product packages were presented. For imported milk samples, liking scores decreased when consumers were aware of the product information and that it was imported milk. However, no significant difference was noted in the change in liking scores depending on whether product package and information was disclosed (*p* < 0.05). Only KEC1 showed a significant increase from 6.87 to 7.25 (*p* < 0.05) in overall liking after presenting the product brand.

The mean acceptance ratings of the appearance, aroma, taste/flavor, and mouthfeel liking of the samples for the blind and informed tests are provided in Table 3. Domestically produced refrigerated milk samples (KEC1 and KEC2) received the highest liking, followed by domestically produced sterilized milk samples (KER1 and KER2). Imported sterilized milk received relatively low liking ratings (DER, PLR, and AUR). The mean liking ratings were increased when the brand information was disclosed for domestic samples. Conversely, the mean liking ratings decreased for imported samples when the brand information was disclosed. For most of the samples, there was no significant difference in liking ratings. However, similar to overall liking, the other liking ratings for domestic milk increased in the informed test, while those for imported milk decreased.

**Table 3:** Mean acceptance ratings of appearance, aroma, taste/flavor and mouthfeel likings of milk samples for the blind and informed tests

Samples	Appearance			Aroma			Taste/flavor			Mouthfeel		
	B^1)^	I	*P*-value^2)^	B	I	*P*-value	B	I	*P*-value	B	I	*P*-value
KEC1	7.11^a3)^	7.11^a^	n.s.	6.39^a^	6.76^a^	n.s.	6.91^a^	7.33^a^	*	6.73^a^	7.10^a^	*
KEC2	6.84^a^	6.75^a^	n.s.	6.26^ab^	6.47^ab^	n.s.	6.65^ab^	6.84^ab^	n.s.	6.61^a^	6.78^ab^	n.s.
KER1	6.60^ab^	6.65^a^	n.s.	5.66^bcd^	5.90^b^	n.s.	5.47^cd^	5.86^c^	n.s.	5.91^bc^	6.14^c^	n.s.
KER2	6.83^a^	6.76^a^	n.s.	5.78^abc^	6.08^b^	n.s.	6.15^bc^	6.32^bc^	n.s.	6.39^ab^	6.35^bc^	n.s.
AUR	5.74^c^	5.37^b^	*	5.44^cd^	5.13^c^	n.s.	4.88^de^	4.33^d^	*	5.60^cd^	5.25^d^	n.s.
PLR	6.16^bc^	5.87^b^	n.s.	5.07^de^	4.77^cd^	n.s.	4.60^e^	4.33^d^	n.s.	5.40^cd^	5.11^d^	n.s.
DER	6.12^bc^	5.87^b^	n.s.	4.72^e^	4.40^d^	n.s.	3.80^f^	3.57^e^	n.s.	5.18^d^	4.81^d^	n.s.

^1)^ B: blind test, I: informed test

^2)^ Paired samples t-test for acceptance between test conditions; (*) *P* < 0.05; n.s. means non-significant

^3)^ Different superscripts within a column indicate significant difference at *P* < 0.0001 by Tukey’s honest significant difference test

**Fig. 4:**
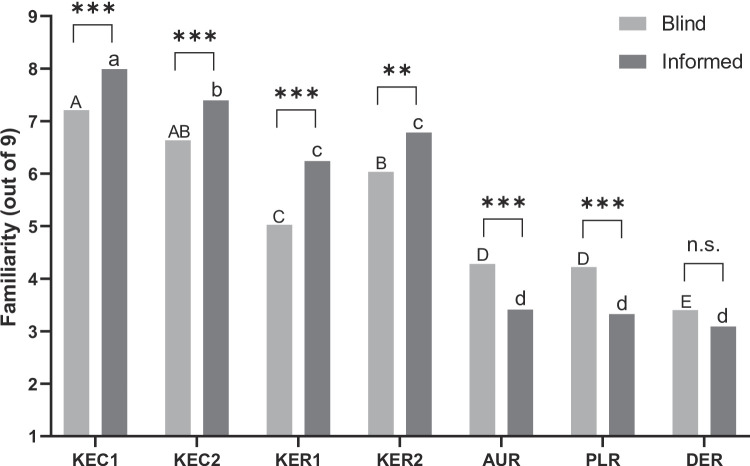
Comparison of mean familiarity ratings of the blind and informed tests of milk samples. Uppercase letters indicate significant differences (*p* < 0.001) among samples in the blind test, whereas lowercase letters indicate significant differences in the informed test. ** and *** means a significant difference between the blind and informed tests for each same sample at *p* < 0.01, and *p* < 0.001, respectively. n.s. meant non-significant

The familiarity scores of the samples in the blind and informed tests are presented in Fig. 4. Significant differences in familiarity were noted across the samples (*p* < 0.05), and the same mean separation pattern was found between the blind and informed tests. Domestically produced refrigerated milk samples showed the highest familiarity (KEC1 and KEC2), followed by domestically produced sterilized milk samples (KER1 and KER2). Imported sterilized milk received relatively low liking ratings (DER, PLR, and AUR). When the product package and information was disclosed, the familiarity of domestic samples increased significantly (*p* < 0.05). However, the familiarity dropped for imported samples(*p* < 0.05), except for DER, which had the lowest familiarity.

### Comparison of the sensory profiles between the blind and informed test

**Fig. 5:**
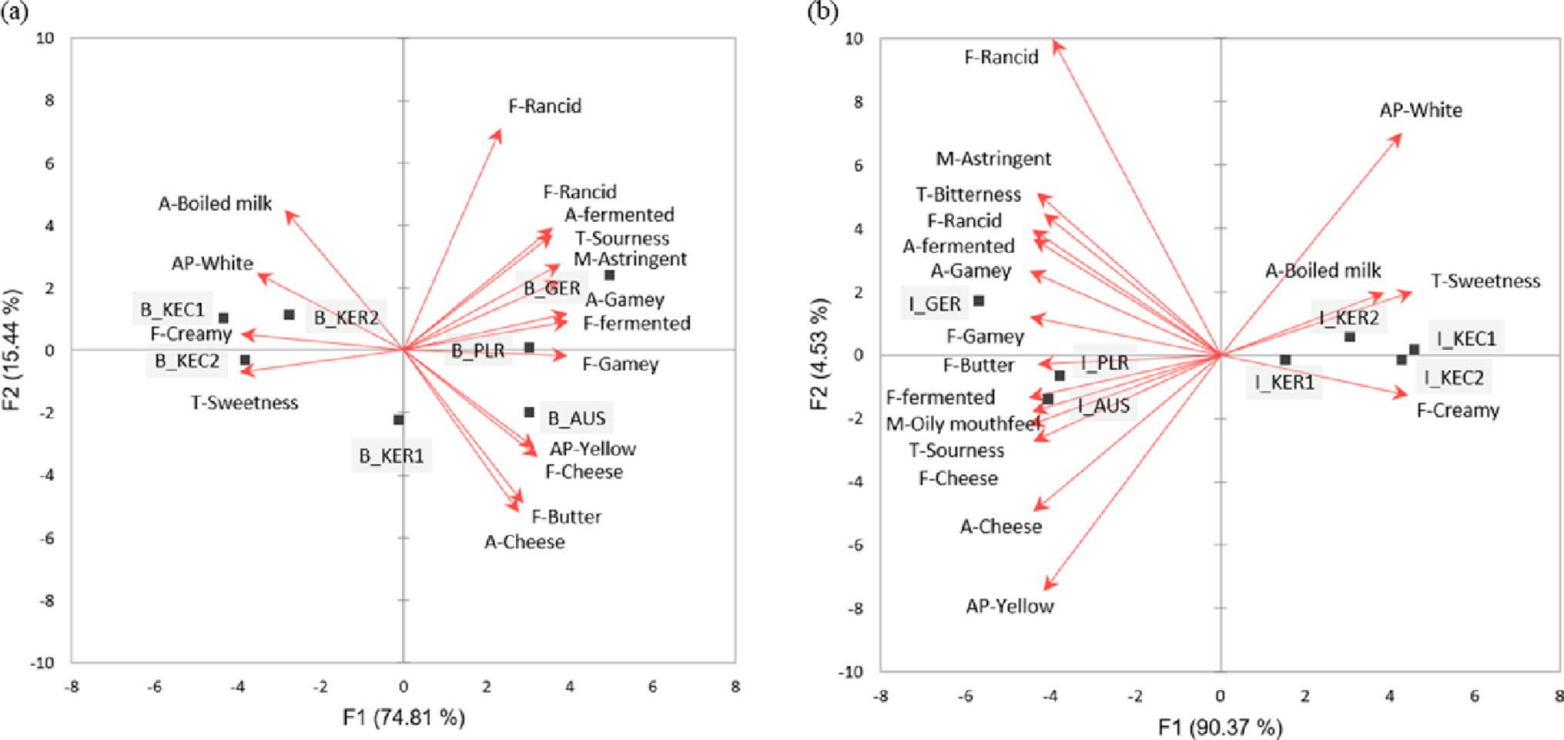
PCA biplots for overall acceptance of milk samples obtained from the (**a**) blind and (**b**) informed tests. Squares and arrows represent samples and sensory attributes, respectively. Sample codes are given in Table 1. The letters ‘B’ and ‘I’ preceding the sample codes indicate the blind and informed tests, respectively. AP, A, F, T, and M in front of each sensory attribute mean appearance, aroma, flavor, taste, and mouthfeel, respectively.

The mean intensity ratings of the 25 attributes for the blind and informed tests are shown in Supplementary Table S3. Significant differences were observed in 16 attributes in both blind and informed tests, whereas 7 attributes (opaque appearance, creamy aroma, saltiness, boiled milk flavor, viscosity, residual perception, and viscosity mouthfeel) showed no significant differences, and 2 attributes (bitterness and oily mouthfeel) demonstrated significant differences among the samples only in the informed test. The PCA biplots for sensory attributes from the blind and informed tests are shown in Fig. 5. The blind test (Fig. 5a showed a total variance of 90.25% (F1, 74.81%; F2, 15.44%), and clearly distinguished between domestic and imported milk samples. Overall, domestic milk was characterized by a white appearance, boiled milk aroma, sweetness, and creamy flavor. Conversely, imported milk appeared yellow and was notably associated with intense dairy-related aromas such as cheese, butter, fermented and a gamey aromas and flavors. Imported milk samples were also found to have a more yellowish color. Extrinsic factors of milk, such as the product package and price, appear to have slight influence on the sensory profiling. The informed test showed a total variance of 94.90% (F1, 90.37%; F2, 4.53%), and the sensory characteristics of domestic and imported milk became more distinct (Fig. 5b). The sensory characteristics of the samples were similar to the results from the informed test (Fig. 5a; however, domestic and imported samples were more clearly divided and grouped. These findings suggest that extrinsic factors exert little influence on the evaluation of sensory attributes. Several studies also reported that the same samples showed similar sensory profiles across different test locations (Kim et al., 2023; Niimi et al., 2022; Oh et al., 2024; Park et al., 2023, 2024) or between blind versus informed test conditions (de Andrade et al., 2018; Grasso et al., 2022; Kwak et al., 2017).

### Correlation of volatile aroma compounds and sensory profiling in the blind and informed test

**Fig. 6:**
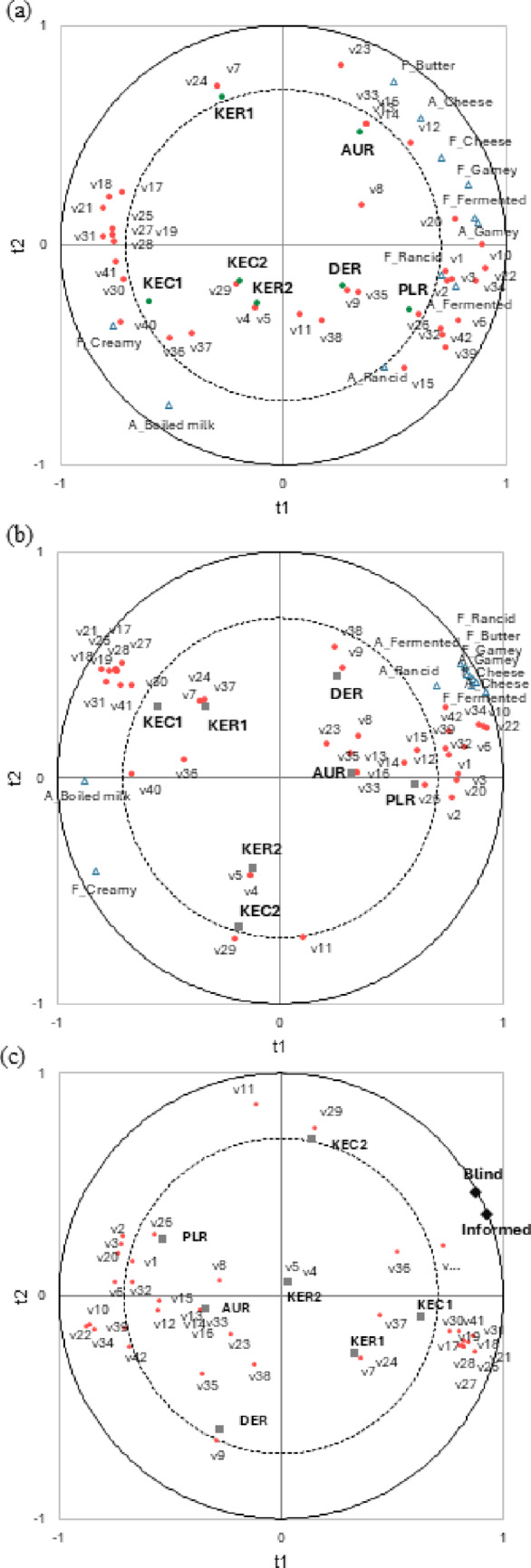
Partial least squares regression analysis using 42 volatile aroma compounds (independent variables) and sensory evaluation results (dependent variables); (**a**) aroma and flavor from the blind test, (**b**) aroma and flavor from the informed test, (**c**) overall liking ratings from blind and informed test. Each volatile aroma compound (v1-v42) refers to Supplementary Table S1

PLS regression analyses of volatile aroma compounds and sensory profile ratings of aroma and flavor and overall liking are shown in Fig. 6. Figure 6a showed the PLS results between volatile aroma compounds and aroma and flavor ratings from the blind test. The volatile aroma compounds clearly differentiated between domestic and imported milk samples. Imported milk was strongly associated with sensory attributes such as cheese, butter, fermented, gamey, and rancid characteristics. Specifically, DER and PLR exhibited strong rancid, fermented, and gamey characteristics, which were highly correlated with compounds such as hexanal, 3-hydroxybutan-2-one, nonan-2-one, undecan-2-one, methylsulfonylmethane, butan-2-one, propan-2-one, and pentan-2-one. While AUR showed stronger cheesy and buttery attributes, these characteristics were not clearly associated with specific volatile compounds in the analysis. In contrast, domestic milk samples were located on the opposite side of the plot, as they either lacked or had significantly lower intensities of the aroma attributes prominent in imported milk. Instead, they showed some association with compounds related to sweet and aromatic characteristics, such as 1,2-xylene and phenylmethanol. Figure 6b showed the PLS results between volatile aroma compounds and aroma and flavor ratings from the informed test. Consumers evaluated the sensory characteristics of the samples with product information, leading to more distinct differentiation of sensory profiles based on package information. Among domestic milk samples, KEC1 and KER1, which have relatively strong aroma and flavor, showed high correlation with compounds such as 1,2-xylene and phenylmethanol (associated with sweet and aromatic notes) and many of volatile aroma compounds were not directly related to milk. In contrast, KEC2 and KER2, which showed weaker aroma and flavor characteristics, were positioned on the opposite side of the plot, away from the volatile aroma compounds. When consumers recognized the samples as imported milk, they perceived stronger characteristics such as cheese, butter, fermented, gamey, and rancid attributes. These characteristics became more clear in the informed test when consumers were aware of the product information, causing dairy-related attributes to cluster more closely in the same direction on the correlation map (Fig. 6b) compared to the blind test (Fig. 6a. However, despite these associations, it was difficult to clearly differentiate the sensory characteristics among the imported milk samples. Figure 6c showed the PLS results between volatile aroma compounds and overall liking from the blind and informed tests. The results clearly showed that aroma and flavors related to fermented, cheese, yogurt, butter, and gamey attributes were related to imported milk samples such as propan-2-one, butan-2-one, pentan-2-one, heptan-2-one, nonan-2-one, undecan-2-one, and methylfulfanylmethane (Table S1) and these volatile aroma compounds influence negatively to the overall liking. Domestic samples had relatively weaker sensory profile for these attribute (Table S3) and have lower relative contents of the volatile aroma compounds (Fig. 1).

## Discussion

In this study, the differences in the volatile aroma compounds of Korean and imported milk significantly influenced consumer acceptance, familiarity, and sensory profiles. Imported milk samples had relatively higher contents of volatile aroma compounds (Fig. 1) and detailed aroma profiles (Tables S1 and S2). Domestic milk showed a lower content of volatile aroma compounds compared to the imported milk in general. The analysis of volatile aroma compounds was closely associated with the sensory perception of milk, and might be particularly associated with the gamey odor (*nurinnae*) that has been frequently mentioned in relation to imported milk. The unfamiliar flavor of the imported milk appeared to result in lower acceptance ratings than Korean milk. Due to Korea’s limited land area and high population density, dairy cows are raised in barns and primarily fed formulated fodder, resulting in milk with a milder, less complex aroma. In contrast, pasture-based feeding, common in some imported milk-producing countries, contributes to richer and sometimes more intense flavor profiles (Chi et al., 2023). Korean consumers, who are generally accustomed to milk with a subtle aroma and flavor (Chung, 2009), likely perceive imported milk with a stronger aroma and flavor derived from pasture-fed cows as unpleasant. These findings are in line with the differences in fatty acid compositions, which have a critical influence on flavor and taste, between Korean beef raised on grain feed and Australian beef raised on pasture (Jo et al., 2012). Korean consumers have an acquired taste for domestically produced milk due to long-term consumption (Havermans & van den Heuvel, 2023). Having been exposed to domestic milk for an extended period, Korean consumers are likely more accustomed to its mild aroma and flavor characteristics. In contrast, imported milk has only been available in the Korean market for a few years, meaning that consumers have had limited exposure to its distinct taste and aroma. As a result, the unfamiliarity with the flavor characteristics of imported milk may have contributed to its lower acceptance and familiarity among Korean consumers. This aligns with previous research indicating that consumer perceptions of dairy products are influenced by long-term exposure and cultural familiarity, which influence taste preferences over time (Baran et al., 2024). The differences in volatile chemical compounds were further confirmed by the PLS regression analysis between volatile chemical compounds and sensory evaluation results. Imported milk showed strong dairy-related sensory attributes, such as fermented, butter, cheese, and yogurt attributes, which showed strong correlations with specific volatile chemical compounds (Fig. 6a, b). These characteristics negatively influenced consumer liking ratings (Fig. 6c. The dairy-related attributes in milk were generally perceived negatively by Korean consumers, leading to decreased overall liking.

Korean milk received higher acceptance ratings in the blind test, which further increased in the informed test when package and price information were provided for the most familiar milk sample (KEC1). This indicates that consumer ethnocentrism, where familiarity and trust in domestic products are effective in the increasing preference to some degree. Increasing overall acceptance and familiarity indicated that extrinsic information has additional effects on consumer preferences. This finding is consistent with Büttner et al. (2015), who showed that the presence of product information, including brand and origin, substantially influenced consumer evaluations. Similarly, Balabanis and Diamantopoulos (2004) demonstrated that consumers tended to prefer domestic products due to perceived higher quality and trustworthiness. Also, COO strongly influences perceived quality and purchase intentions (Magnier et al., 2016). The increased acceptance for Korean milk when the brand and COO were known emphasize the strong consumer trust in domestic products, which has been reinforced by years of local branding and quality assurance (Lee & Yoo, 2017).

In contrast, imported milk showed lower acceptance ratings in the blinded test, and its acceptance further decreased when product information was provided. In the blind test, imported milk received lower acceptance based on its intrinsic quality such as strong dairy-related aroma, and the acceptance declined slightly more in the informed test when product information was provided. Deliza et al. (2005) noted that extrinsic cues, such as COO, package, and brand, can significantly influence consumer perceptions. Consumers often rely on these cues to make judgments, particularly when the product is less familiar, resulting in decreased preferences for foreign products. Magnier and Schoormans (2017) highlighted that packaging and labeling cues, such as the brand, color and production origin, can influence consumer perceptions and drive purchasing decisions. Therefore, unfamiliarity of extrinsic cues in imported milk generated additionally lower acceptance rating in the informed test. These results underscore the importance of the effect of the COO on consumer preferences, particularly in Korea, where domestic products hold significant cultural and psychological value.

Price was expected to influence the informed test results; however, its actual effect was minimal. Given the high price of Korean milk, the lower price of imported milk might influence an increase in acceptance for samples such as PLR. Intrinsic factors, such as flavor and taste, were more influential in the consumer acceptance of milk since consumers are sensitive to milk price. Our findings indicate that the sensory quality of milk is the dominant factor driving consumer acceptance although consumers may be sensitive to milk price. Despite Polish milk being nearly 46% cheaper than Korean milk, no notable difference was noted in the acceptance ratings between the blind and informed tests. Only few participants commented in open-ended responses that they preferred the milk because of its lower price (Results not shown). For the other two imported products with smaller price differences, the minimal effect of price was observed. Even though milk price in Korea is high, milk remains a relatively inexpensive product compared with items such as cars or home appliances, which have a more significant influence on household finances. Therefore, price seemed not to be a major consideration for milk consumption.

The familiarity with domestic milk significantly increased across all samples in the informed test, whereas familiarity with imported milk decreased in all samples (Table 3). These changes in familiarity were more pronounced when package and price information were presented, compared to when consumers judged the samples based solely on intrinsic factor; aroma, taste and flavor. Familiarity with domestic milk, already well-established among Korean consumers, further increased with product information. In contrast, the perception of imported milk as UHT-treated had diminished familiarity because of its distinct taste and aroma compared with conventional milk in Korea. This finding indicates that consumer perceptions of imported milk negatively influenced familiarity, even if imported milk was more price-competitive than domestic milk. Over time, Korean consumers seemed to have acquired a taste for Korean milk, which has different volatile aroma compounds and sensory profiles.

In the informed test, both product packaging and price were presented to the participants. They could easily compare the prices displayed on the packaging. Although various media in Korea have reported the cost-effectiveness of imported milk (ChosunBiz, 2024; Hankyung, 2024), the participants in this study demonstrated decreased acceptance and familiarity for imported milk, even when they were aware of its price competitiveness. This is the supporting evidence that the negative intrinsic factors such as aroma and taste, along with extrinsic cues such as COO, outweigh the benefits of a lower price. Although imported milk was offered at an average of approximately 21% lower price than domestic milk in this study, consumers appeared to prioritize the intrinsic factors of milk, such as aroma, flavor, and taste, over price by looking at the difference of ratings in the informed test. Although the amount of imported milk has increased, the limited consumption experience of imported milk and its relatively low prevalence in the domestic market may contribute to consumers’ lack of familiarity with it.

## Conclusions

Domestic and imported milk showed distinctive differences in volatile aroma compounds. The volatile aroma compounds detected in milk samples were predominantly fatty acid-derived ketones and benzene derivatives, with imported milk containing significantly higher levels of these compounds. In particular, sulfur compounds, known for their low threshold level and distinct aroma, were detected at relatively higher concentrations in imported milk. Therefore, the high concentrations of distinctive volatile aroma compounds in imported milk, such as sulfur-containing compounds, might significantly influence consumer preferences. The consumer test demonstrates that intrinsic factors, such as flavor and aroma, significantly influence consumer acceptance of milk, whereas extrinsic factors such as packaging and price showed limited effects. Korean consumers exhibited strong ethnocentric preferences, favoring domestic milk because of its familiarity and milder flavor profile. However, imported milk, characterized by stronger dairy-related aromas, faced challenges in acceptance due to both sensory unfamiliarity and negative perceptions triggered by product information. Despite its price advantage, the strong dairy-related and off- flavors and aromas of imported milk were negative factors in Korean consumers’ preference. Although the volume of imported milk is increasing, Korean consumers have become accustomed to the taste of domestic milk, and it appears that they do not prefer the intense dairy-related aroma of imported milk. Further studies are necessary to compare domestic and imported milk in beverage matrices, such as flavored milk or lattes, which can mask the intense dairy-related aromas.

## Supplementary Information

Below is the link to the electronic supplementary material.

Supplementary Material 1
