# Supplementary material for: Comparison of volatile aroma compounds and consumer perception between domestic and imported milk in Korea
Source: Food Sci Anim Resour. 2026 Apr 2;46(1):52. doi: 10.1007/s44463-025-00018-9 (PMC13046923; doi:10.1007/s44463-025-00018-9)
Supplement: Supplementary file 1 — Supplementary Material 1 [file 44463_2025_18_MOESM1_ESM.docx]

**Supplementary materials**

**Supplementary Table S1.** Identified volatile aroma compounds derived from milk samples

| No. ^1)^ | Volatile compound | RI^2)^ | Flavor description^6)^ | Contents (Mean±SD) | | | | | | |
| --- | --- | --- | --- | --- | --- | --- | --- | --- | --- | --- |
|  |  |  |  | KEC1^3)^ | KEC2 | KER1 | KER2 | AUR | PLR | DER |
| **Acid** |  |  |  |  |  |  |  |  |  |  |
| v35 | acetic acid | 1,477 | Pungent, sour | ND^a4),5)^ | ND^a^ | 0.28±0.04^b^ | 0.45±0.04^d^ | ND^a^ | 0.35±0.05^c^ | 0.35±0.02^c^ |
| **Aldehyde** | |  |  |  |  |  |  |  |  |  |
| v15 |  | 1,089 | Fatty-green | 1.65±0.06^c^ | 0.94±0.02^b^ | ND^a^ | 1.06±0.02^b^ | 1.66±0.13^c^ | 2.13±0.18^d^ | 1.72±0.02^c^ |
| **Alcohol** | |  |  |  |  |  |  |  |  |  |
| v7 | propan-2-ol | 938 | Rubber-alcohol | ND^a^ | ND^a^ | 0.4±0^b^ | ND^a^ | ND^a^ | ND^a^ | ND^a^ |
| v8 | ethanol | 946 | Alcoholic | ND^a^ | ND^a^ | 1.66±0.11^b^ | ND^a^ | ND^a^ | 2.31±0.19^c^ | ND^a^ |
| v26 | 3-methylbutan-1-ol | 1,224 | Winey-brandy | ND^a^ | ND^a^ | ND^a^ | ND^a^ | ND^a^ | 0.78±0.06^b^ | ND^a^ |
| v33 | hexan-1-ol | 1,370 | Chemical, Slight fatty-fruity | ND^a^ | ND^a^ | ND^a^ | ND^a^ | 0.11±0^b^ | ND^a^ | ND^a^ |
| v36 | 2-ethylhexan-1-ol | 1,507 | Weak rose-like | 1.12±0.03^d^ | 0.4±0.04^c^ | 0.1±0.01^a^ | 0.27±0.05^b^ | 0.32±0.03^b^ | 0.42±0.05^c^ | 0.14±0.01^a^ |
| v40 | 2-(2-ethoxyethoxy)ethanol | 1,642 | - | 0.27±0.04^c^ | 0.12±0.01^b^ | ND^a^ | ND^a^ | ND^a^ | ND^a^ | ND^a^ |
| **Ketones** | |  |  |  |  |  |  |  |  |  |
| v3 | propan-2-one | 816 | Characteristic solvent odor | 20.12±1.32^b^ | 20.66±0.48^b^ | 5.82±0.25^a^ | 5.57±0.2^a^ | 46.53±1.69^c^ | 78.75±5.19^d^ | 22.79±0.84^b^ |
| v6 | butan-2-one | 907 | Sweet, solvent, fruity camphor odor | 6.64±0.4^b^ | 7.76±0.49^b^ | 0.68±0.05^a^ | 1.29±0.02^a^ | 15.55±0.43^c^ | 61.6±4.07e | 26.7±1^d^ |
| v10 | pentan-2-one | 983 | Ethereal fruity-ketonic, rum & whiskey odor | 4.15±0.23^c^ | 2.88±0.18^bc^ | 0.94±0.08^a^ | 1.95±0.04^ab^ | 26.23±0.87e | 28.97±1.93f | 18.42±0.66^d^ |
| v14 | 3-methyl-5-propylnonane | 1,055 | - | ND^a^ | ND^a^ | ND^a^ | ND^a^ | 2.07±0.11^b^ | ND^a^ | ND^a^ |
| v22 | heptan-2-one | 1,189 | Fruity, cheese, cinnamon odor | 12.05±0.77^b^ | 9.6±0.88^b^ | ND^a^ | 8.82±0.35^b^ | 62.4±2.34^d^ | 77.95±5.14e | 54.18±2.11^c^ |
| v32 | 3-hydroxybutan-2-one | 1,297 | Yogurt-like | ND^a^ | ND^a^ | ND^a^ | ND^a^ | ND^a^ | 1.22±0.13^c^ | 0.39±0.04^b^ |
| v34 | nonan-2-one | 1,399 | Fatty-cheese | 2.25±0.1^c^ | 1.62±0.05^b^ | 0.19±0.01^a^ | 1.39±0.02^b^ | 6.4±0.06e | 7.52±0.53f | 5.91±0.15^d^ |
| **Aliphatic hydrocarbons** | |  |  |  |  |  |  |  |  |  |
| v2 | 2,3,3-trimethylpentane | <800 | - | ND^a^ | ND^a^ | ND^a^ | 3.37±0.1^b^ | 8.02±0.13^c^ | 22.22±1.49^d^ | ND^a^ |
| v9 | 2,2,4,6,6-pentamethylheptane | 947 | - | ND^a^ | ND^a^ | ND^a^ | ND^a^ | ND^a^ | ND^a^ | 0.59±0.06^b^ |
| v11 | decane | 1,001 | Gasoline-like | ND^a^ | 1.19±0.01^c^ | ND^a^ | ND^a^ | ND^a^ | 0.56±0.08^b^ | ND^a^ |
| v13 | 2,2,3,4-tetramethylpentane | 1,030 | - | ND^a^ | ND^a^ | ND^a^ | ND^a^ | 5±0.01^b^ | ND^a^ | ND^a^ |
| v39 | undecan-2-one | 1,613 | Waxy-fruity | ND^a^ | ND^a^ | ND^a^ | 0.15±0^b^ | ND^a^ | 0.86±0.11^d^ | 0.59±0.04^c^ |

**Supplementary Table S1**. Cont.

| No.^a^ | Volatile compound | RI^b^ | Flavor description^6)^ | Contents (Mean±SD) | | | | | | |
| --- | --- | --- | --- | --- | --- | --- | --- | --- | --- | --- |
|  |  |  |  | KEC1 | KEC2 | KER1 | KER2 | AUR | PLR | DER |
| **Benzenes** | |  |  |  |  |  |  |  |  |  |
| v17 | ethylbenzene | 1,125 | Gasoline-like | 5.53±0.32e | 0.9±0.02^bc^ | 4.19±0.37^d^ | 0.43±0.05^a^ | 1±0.16^c^ | 1.06±0.12^c^ | 0.54±0.06^ab^ |
| v18 | 1,4-xylene | 1,132 | Plastic-like | 6.98±0.42^d^ | 1.32±0.02^b^ | 5.16±0.65^c^ | 0.58±0.04^a^ | 1.02±0.05^ab^ | 0.79±0.1^ab^ | 0.65±0.06^a^ |
| v19 | 1,3-xylene | 1,138 | Plastic-like | 25.83±1.71^d^ | 2.82±0.18^b^ | 13.46±1.62^c^ | 1.03±0.03^a^ | 2.27±0.11^ab^ | 2.11±0.18^ab^ | 1.61±0.02^ab^ |
| v21 | 1,2-xylene | 1,183 | Mild sweet odor | 12.74±0.81e | 1.32±0.02^c^ | 9.02±0.29^d^ | 0.69±0.04^b^ | ND^a^ | ND^a^ | 0.9±0.05^bc^ |
| v24 | propylbenzene | 1,214 | - | ND^a^ | ND^a^ | 0.18±0.03^b^ | ND^a^ | ND^a^ | ND^a^ | ND^a^ |
| v25 | 1-ethyl-4-methylbenzene | 1,223 | - | 1.46±0.05^c^ | ND^a^ | 0.66±0.04^b^ | ND^a^ | ND^a^ | ND^a^ | ND^a^ |
| v27 | 1-ethyl-2-methylbenzene | 1,225 | - | 2.22±0.1^c^ | ND^a^ | 1.01±0.12^b^ | ND^a^ | ND^a^ | ND^a^ | ND^a^ |
| v28 | 1,3,5-trimethylbenzene | 1,245 | Earthy-musty | 1.33±0.04^c^ | ND^a^ | 0.55±0.03^b^ | ND^a^ | ND^a^ | ND^a^ | ND^a^ |
| v29 | styrene | 1,263 | Resinous | ND^a^ | 1.2±0.01^b^ | ND^a^ | ND^a^ | ND^a^ | ND^a^ | ND^a^ |
| v30 | 1-ethyl-3-methylbenzene | 1,264 | Pungent solvent | 1.26±0.04^c^ | ND^a^ | 0.22±0.03^b^ | ND^a^ | ND^a^ | ND^a^ | ND^a^ |
| v31 | 1,2,4-trimethylbenzene | 1,284 | - | 4.15±0.23^d^ | 0.32±0.08^b^ | 2.02±0.17^c^ | 0.21±0.05^ab^ | ND^a^ | ND^a^ | ND^a^ |
| v37 | benzaldehyde | 1,537 | Sweet cherry | 1.63±0.06e | 0.6±0.05^b^ | 0.48±0.06^a^ | 0.61±0.04^b^ | 0.73±0.02^c^ | 0.84±0.11^d^ | 0.7±0.06^bc^ |
| v41 | phenylmethanol | >1800 | Faint sweet | 1.87±0.07^d^ | 0.25±0.01^ab^ | 0.82±0.09^c^ | 0.34±0.05^b^ | 0.19±0.02^a^ | 0.34±0.02^b^ | 0.18±0.01^a^ |
| **Cyclic hydrocarbons** | |  |  |  |  |  |  |  |  |  |
| v4 | 1,1,3-trimethylcyclohexane | 863 | - | ND^a^ | ND^a^ | ND^a^ | 1.01±0.03^b^ | ND^a^ | ND^a^ | ND^a^ |
| v5 | ethylcyclohexane | 889 | - | ND^a^ | ND^a^ | ND^a^ | 1.54±0.03^b^ | ND^a^ | ND^a^ | ND^a^ |
| **Terpenes** | |  |  |  |  |  |  |  |  |  |
| v12 | Alpha-pinene | 1,025 | Fresh, sweet | ND^a^ | ND^a^ | ND^a^ | ND^a^ | 3.87±0.04^d^ | 0.98±0.11^c^ | 0.41±0.04^b^ |
| v16 | Beta-pinene | 1,105 | Woody | ND^a^ | ND^a^ | ND^a^ | ND^a^ | 2.15±0.11^b^ | ND^a^ | ND^a^ |
| v20 | DELTA.3-Carene | 1,148 | Fresh, sweet | ND^a^ | ND^a^ | ND^a^ | ND^a^ | 0.77±0.08^b^ | 0.94±0.11^c^ | ND^a^ |
| v23 | Limonene | 1,202 | Citrus | ND^a^ | ND^a^ | 0.11±0.01^b^ | ND^a^ | 0.29±0.01^c^ | ND^a^ | ND^a^ |
| **Sulfur-compounds** | |  |  |  |  |  |  |  |  |  |
| v1 | methylsulfanylmethane | <800 | Pungent, cabbage, cooked vegetable odor | 2.09±0.09^b^ | ND^a^ | 2.66±0.15^b^ | ND^c^ | 8.5±0.15^a^ | 26.47±1.77^d^ | 3.03±0.04^b^ |
| v38 | methylsulfinylmethane | 1,601 | Fatty, salty, garlic | 0.55±0.03e | ND^a^ | ND^a^ | ND^a^ | 0.27±0.01^b^ | 0.42±0.04^d^ | 0.35±0.02^c^ |
| v42 | methylsulfonylmethane | >1800 | Sulfurous burnt | ND^a^ | ND^a^ | ND^a^ | ND^a^ | ND^a^ | 0.42±0.04^c^ | 0.35±0.02^b^ |

^1)^ Numbered as in the order of retention indices (RI).

^2)^ Retention indices were determined using n-paraffins C7–C22 as external standards.

^3)^ Sample codes referred to Table 1.

^4)^ There are significant differences (p<0.05) among fish cake samples by using Duncan’s multiple comparison.

^5)^ Not detected.

^6)^ Odor description found in the commercial library (flavorbase 2010, leffingwell & Associates, Georgia, USA).

**Supplementary Table S2.** Main volatile aroma compounds contributing the difference between the imported and domestic milk samples

| No. | volatile compound | VIP | pcorr | Odor description^1)^ |
| --- | --- | --- | --- | --- |
| Imported milk (positive PLS1) | |  |  |  |
| v22 | heptan-2-one | 1.6 | 1.0 | Fruity, cheese, cinnamon odor |
| v10 | pentan-2-one | 1.6 | 0.9 | Ethereal fruity-ketonic, rum & whiskey odor |
| v34 | nonan-2-one | 1.6 | 0.9 | Fruity, fatty-cheese-herbal-coconut odor |
| v3 | propan-2-one | 1.3 | 0.8 | Characteristic solvent odor |
| v20 | alpha-pinene | 1.3 | 0.8 | Fresh, sweet |
| v6 | butan-2-one | 1.3 | 0.8 | Sweet, solvent, fruity camphor odor |
| v2 | 2,3,3-trimethylpentane | 1.1 | 0.8 | - |
| v1 | methylsulfanylmethane | 1.1 | 0.8 | Pungent, cabbage, cooked vegetable odor |
| Domestic milk (Negative PLS2) | |  |  |  |
| v21 | 1,2-xylene | 1.0 | -0.8 | mild sweet odor |
|  |  |  |  |  |

^1)^ Odor description found in the commercial library (flavorbase 2010, leffingwell & Associates, Georgia, USA).

**Supplementary Table S3.** Mean intensity ratings of 25 sensory attributes of milk samples for blind and informed tests.

| Attributes | Blind | | | | | | | | Informed | | | | | | | |
| --- | --- | --- | --- | --- | --- | --- | --- | --- | --- | --- | --- | --- | --- | --- | --- | --- |
|  | KEC1^1)^ | KEC2 | KER1 | KER2 | AUS | PLR | GER | p-value | KEC1 | KEC2 | KER1 | KER2 | AUS | PLR | GER | p-value |
| **Appearance** |  |  |  |  |  |  |  |  |  |  |  |  |  |  |  |  |
| White | 2.55^a2)^ | 2.43^a^ | 2.14^b^ | 2.35^ab^ | 1.10^d^ | 1.54^c^ | 1.69^c^ | *** | 2.54^a^ | 2.42^a^ | 2.23^a^ | 2.38^a^ | 1.19^c^ | 1.42^bc^ | 1.64^b^ | *** |
| Yellow | 0.32^d^ | 0.38^d^ | 0.54^cd^ | 0.40^d^ | 0.65^a^ | 1.10^b^ | 0.86^bc^ | *** | 0.29^c^ | 0.32^c^ | 0.47^c^ | 0.33^c^ | 1.72^a^ | 1.25^b^ | 1.13^b^ | *** |
| Opaque | 0.61 | 0.80 | 0.63 | 0.72 | 0.61 | 0.66 | 0.64 | n.s. | 0.59 | 0.61 | 0.65 | 0.68 | 0.58 | 0.61 | 0.61 | n.s. |
| **Aroma** |  |  |  |  |  |  |  |  |  |  |  |  |  |  |  |  |
| Creamy | 0.82 | 0.90 | 1.13 | 0.84 | 0.86 | 1.06 | 0.95 | n.s. | 0.66 | 0.75 | 0.89 | 0.70 | 0.90 | 0.98 | 0.81 | n.s. |
| Cheese | 0.67^c^ | 0.85^bc^ | 1.39^a^ | 0.75^c^ | 1.20^ab^ | 1.39^a^ | 1.03^abc^ | *** | 0.47^c^ | 0.51^c^ | 0.79^bc^ | 0.53^c^ | 1.19^a^ | 1.13^ab^ | 1.08^ab^ | *** |
| Boiled milk | 1.00^a^ | 0.82^bc^ | 0.70^ab^ | 0.96^ab^ | 0.58^b^ | 0.85^ab^ | 0.69^ab^ | * | 0.97^a^ | 0.84^a^ | 1.01^a^ | 0.97^a^ | 0.67^b^ | .076^b^ | 0.68^b^ | * |
| Fermented | 0.40^b^ | 0.37^b^ | 0.47^ab^ | 0.51^ab^ | 0.54^ab^ | 0.70^ab^ | 0.81^ab^ | ** | 0.32^bc^ | 0.25^c^ | 0.37^bc^ | 0.36^bc^ | 0.59^ab^ | 0.70^a^ | 0.81^a^ | *** |
| Rancid | 0.65^ab^ | 0.56^b^ | 0.51^b^ | 0.64^ab^ | 0.56^b^ | 0.76^ab^ | 0.97^a^ | ** | 0.39^b^ | 0.41^b^ | 0.46^b^ | 0.53^b^ | 0.56^ab^ | 0.64^ab^ | 0.88^a^ | *** |
| Gamey | 0.29^c^ | 0.30^c^ | 0.43^bc^ | 0.42^bc^ | 0.70^ab^ | 0.68^ab^ | 0.82^a^ | *** | 0.20^b^ | 0.17^b^ | 0.29^b^ | 0.26^b^ | 0.72^a^ | 0.78^a^ | 0.99^a^ | *** |
| **Taste** |  |  |  |  |  |  |  |  |  |  |  |  |  |  |  |  |
| Sweetness | 1.43^ab^ | 1.46^a^ | 1.27^abc^ | 1.46^a^ | 1.01^cd^ | 1.04^bcd^ | 0.84^d^ | *** | 1.46^a^ | 1.41^a^ | 1.23^a^ | 1.29^a^ | 0.81^b^ | 0.82^b^ | 0.80^b^ | *** |
| Saltiness | 0.30 | 0.38 | 0.42 | 0.34 | 0.42 | 0.41 | 0.31 | n.s. | 0.32 | 0.28 | 0.27 | 0.31 | 0.41 | 0.31 | 0.30 | n.s. |
| Bitterness | 0.15 | 0.18 | 0.22 | 0.21 | 0.25 | 0.20 | 0.36 | n.s. | 0.12^b^ | 0.13^ab^ | 0.18^ab^ | 0.14^ab^ | 0.26^ab^ | 0.19^ab^ | 0.32^a^ | * |
| Sourness | 0.16^b^ | 0.18^b^ | 0.25^b^ | 0.21^b^ | 0.32^b^ | 0.35^ab^ | 0.51^a^ | *** | 0.12^a^ | 0.14^a^ | 0.21^ab^ | 0.17^a^ | 0.32^b^ | 0.30^b^ | 0.32^b^ | ** |
| **Flavor** |  |  |  |  |  |  |  |  |  |  |  |  |  |  |  |  |
| Cheese | 0.74^c^ | 0.87^bc^ | 1.37^a^ | 0.64^c^ | 1.27^ab^ | 1.60^a^ | 1.24^ab^ | *** | 0.45^d^ | 0.50^d^ | 0.96^bc^ | 0.62^cd^ | 1.27^ab^ | 1.38^a^ | 1.31^ab^ | *** |
| Butter | 0.63^b^ | 0.68^a^ | 1.11^a^ | 0.73^ab^ | 0.97^ab^ | 0.93^ab^ | 0.91^ab^ | ** | 0.56^b^ | 0.56^b^ | 0.83^ab^ | 0.70^ab^ | 0.91^ab^ | 1.03^a^ | 1.01^a^ | *** |
| Boiled milk | 0.98 | 0.83 | 0.75 | 0.93 | 0.61 | 0.86 | 0.78 | n.s. | 0.82 | 0.72 | 0.90 | 0.85 | 0.62 | 0.73 | 0.79 | n.s. |
| Creamy | 1.86^a^ | 1.70^a^ | 1.18^bc^ | 1.61^ab^ | 0.97^cd^ | 1.13^cd^ | 0.70^d^ | *** | 1.91^a^ | 1.72^ab^ | 1.23^cd^ | 1.49^bc^ | 0.88^de^ | 0.87^de^ | 0.59^e^ | *** |
| Fermented | 0.38^bc^ | 0.34^c^ | 0.48^abc^ | 0.45^abc^ | 0.72^ab^ | 0.70^abc^ | 0.80^a^ | *** | 0.25^d^ | 0.25^d^ | 0.37^bcd^ | 0.32^cd^ | 0.63^abc^ | 0.70^a^ | 0.68^ab^ | *** |
| Rancid | 0.49^bc^ | 0.39^c^ | 0.54^bc^ | 0.56^bc^ | 0.66^abc^ | 0.79^ab^ | 1.03^a^ | *** | 0.33^c^ | 0.26^c^ | 0.38^bc^ | 0.34^c^ | 0.71^ab^ | 0.77^a^ | 1.00^a^ | *** |
| Gamey | 0.25^c^ | 0.26^c^ | 0.47^bc^ | 0.37^bc^ | 0.86^a^ | 0.68^ab^ | 0.89^a^ | *** | 0.17^b^ | 0.19^b^ | 0.33^b^ | 0.21^b^ | 0.85^a^ | 1.02^a^ | 1.16^a^ | *** |
| **Mouthfeel** |  |  |  |  |  |  |  |  |  |  |  |  |  |  |  |  |
| Viscosity | 0.77 | 0.80 | 0.72 | 0.79 | 0.75 | 0.69 | 0.73 | n.s. | 0.75 | 0.69 | 0.82 | 0.68 | 0.73 | 0.70 | 0.75 | n.s. |
| Astringent | 0.37^b^ | 0.39^ab^ | 0.51^ab^ | 0.48^ab^ | 0.54^ab^ | 0.65^a^ | 0.72^a^ | * | 0.32^bc^ | 0.28^b^ | 0.39^b^ | 0.39^b^ | 0.60^ab^ | 0.51^ab^ | 0.75^a^ | *** |
| Residual | 1.05 | 1.17 | 1.27 | 1.18 | 1.33 | 1.22 | 1.45 | n.s. | 1.16 | 1.03 | 1.14 | 1.12 | 1.24 | 1.28 | 1.32 | n.s. |
| Mouthcoating | 0.98 | 1.04 | 1.12 | 1.11 | 1.02 | 1.03 | 0.85 | n.s. | 1.07 | 0.92 | 1.07 | 0.87 | 0.96 | 0.96 | 0.94 | n.s. |
| Oily | 0.65 | 0.57 | 0.85 | 0.78 | 0.89 | 0.83 | 0.82 | n.s. | 0.50^ab^ | 0.44^b^ | 0.67^ab^ | 0.47^b^ | 0.86^a^ | 0.80^ab^ | 0.88^a^ | *** |

^1)^ Sample codes referred to Table 1.

^2)^ Different superscripts within a row meant significant difference at p<0.05 by Tukey's honest significance test; (***) p<0.001; (**) p<0.01; (*) p<0.05; n.s. meant non-significant.
